# Pollutant Removal from Synthetic Aqueous Solutions with a Combined Electrochemical Oxidation and Adsorption Method

**DOI:** 10.3390/ijerph15071443

**Published:** 2018-07-09

**Authors:** Amin Mojiri, Akiyoshi Ohashi, Noriatsu Ozaki, Ahmad Shoiful, Tomonori Kindaichi

**Affiliations:** Department of Civil and Environmental Engineering, Graduate School of Engineering, Hiroshima University, 1-4-1 Kagamiyama, Higashihiroshima 739-8527, Japan; amin.mojiri@gmail.com (A.M.); ecoakiyo@hiroshima-u.ac.jp (A.O.); ojaki@hiroshima-u.ac.jp (N.O.); d165199@hiroshima-u.ac.jp (A.S.)

**Keywords:** adsorption, ammonia, electrochemical oxidation, molybdenum, phenols

## Abstract

Eliminating organic and inorganic pollutants from water is a worldwide concern. In this study, we applied electrochemical oxidation (EO) and adsorption techniques to eliminate ammonia, phenols, and Mo(VI) from aqueous solutions. We analyzed the first stage (EO) with response surface methodology, where the reaction time (1–3 h), initial contaminant concentration (10–50 mg/L), and pH (3–6) were the three independent factors. Sodium sulfate (as an electrolyte) and Ti/RuO_2_–IrO_2_ (as an electrode) were used in the EO system. Based on preliminary experiments, the current and voltage were set to 50 mA and 7 V, respectively. The optimum EO conditions included a reaction time, initial contaminant concentration, and pH of 2.4 h, 27.4 mg/L, and 4.9, respectively. The ammonia, phenols, and Mo elimination efficiencies were 79.4%, 48.0%, and 55.9%, respectively. After treating water under the optimum EO conditions, the solution was transferred to a granular composite adsorbent column containing bentonite, limestone, zeolite, cockleshell, activated carbon, and Portland cement (i.e., BAZLSC), which improved the elimination efficiencies of ammonia, phenols, and molybdenum(VI) to 99.9%. The energy consumption value (8.0 kWh kg^−1^ N) was detected at the optimum operating conditions.

## 1. Introduction

Disposal of industrial, agricultural, and municipal waste into lakes and rivers can result in environmental contamination [1], where various pollutants can have detrimental effects on human health. Major aquatic pollutants include ammonia, phenol, and heavy metals. Of these, ammonia nitrogen is one of the most common aquatic pollutants and it contributes to the enhanced eutrophication of rivers and lakes, depletion of dissolved oxygen, and fish toxicity in gaining water [2]. Proposed techniques for eliminating ammonia include air stripping, biological reactors, electrochemical oxidation (EO), ozonation, and adsorption [3,4]. The elimination of ammonia has attracted considerable attention due to the need to control nitrogen pollution and to prevent the eutrophication of water sources.

Meanwhile, phenols are among the most toxic contaminants in wastewater, and phenols and related compounds are prevalent organic contaminants in the wastewater of various chemical plants. Given the widespread prevalence of phenols in wastewater and their toxicity to human and animal life, even at low concentrations, their elimination from wastewater is essential. The efficient elimination of phenols from waste streams has gradually become a major environmental concern [5]. Numerous methods have been applied to phenol elimination in wastewater treatment, including biological treatment, reverse osmosis, adsorption, ion exchange, catalytic oxidation, electrochemical oxidation (EO), and solvent extraction as common conventional methods for eliminating phenols and the related organic substances [6].

Finally, among inorganic pollutants, heavy metals have attracted substantial academic attention. The contamination of water by heavy metals during industrial wastewater disposal is an international environmental issue, as rapid industrialization worldwide has significantly contributed to the release of theoretically toxic heavy metals into aquatic systems [7]. Among heavy metals, Mo is highly toxic. Vital techniques for eliminating metals from water include physical/chemical methods, such as adsorption and EO.

EO is a physical/chemical method for treating water and wastewater, and its application to various types of wastewater has been investigated extensively in recent years. The EO process is a promising wastewater treatment method chiefly due to its effectiveness and the ease of operation [3]. Electrochemical technology, which represents an advanced oxidation process, is the most promising method for treating both organic contaminants and heavy metals. Such technology includes electrodialysis, electrocoagulation, electroflotation, anodic oxidation, and EO [8]. Several researchers have investigated the treatment of different types of wastewater with EO [9]. The process of electrochemical degradation can be divided into direct and indirect oxidation procedures. In direct oxidation, the contaminants are first adsorbed onto the surface of the anode, and then undergo the electron transfer reaction of hydroxyl radical (•OH) formation, which is followed by strong oxidative free radical damage of the contaminant molecular structure, and finally, decomposition into CO_2_. In indirect electrochemical oxidation, to produce strong oxidizing agents (e.g., hypochlorous acid/chlorine, ozone, H_2_O_2_, etc.), oxidation of contaminants and electrochemical oxidation occurs in the bulk solution [10]. For example, Chen et al. [8] investigated EO in the treatment of heavy metal wastewater. They expressed that EO could be effective in removing heavy metals. Meanwhile, Cossu et al. [11] used Ti/PbO_2_ and Ti/SnO_2_ anodes to eliminate ammonia nitrogen and chemical oxygen demand from landfill leachate by EO, and found that EO could remove a large proportion of chemical oxygen demand and ammonia.

Researchers often recommend combined techniques to treat the high pollutant concentrations in industrial wastewater. For example, adsorption is a common wastewater treatment method and is typically combined with other techniques [12]. Several researchers have investigated the use of adsorption to treat various types of wastewater [13,14], and several studies [15] have verified that adsorbents can eliminate considerable amounts of contaminants, especially heavy metals. Numerous adsorbents, such as activated carbon, limestone, shell, cement, and zeolite, have been studied in the literature [3,4]. Here, we used a new composite adsorbent, BAZLSC (i.e., bentonite, zeolite, cockleshell, limestone, activated carbon, and Portland cement) to simultaneously adsorb contaminants and to perform ion exchange.

In this study, we assessed a novel combination of EO and adsorption techniques to achieve 100% elimination efficacy. The aims of this research were to (1) evaluate the performance of a combined system incorporating EO and adsorption to remove ammonia, phenols, and Mo from aqueous solutions, (2) introduce a novel process, granular BAZLSC adsorption combined with EO, and (3) monitor the adsorption isotherms during adsorption treatment. There are no reports in the literature of reactors with the same design as our reactor with such a high performance. In addition, we introduced a novel composite adsorbent.

## 2. Materials and Methods

**Figure 1:**
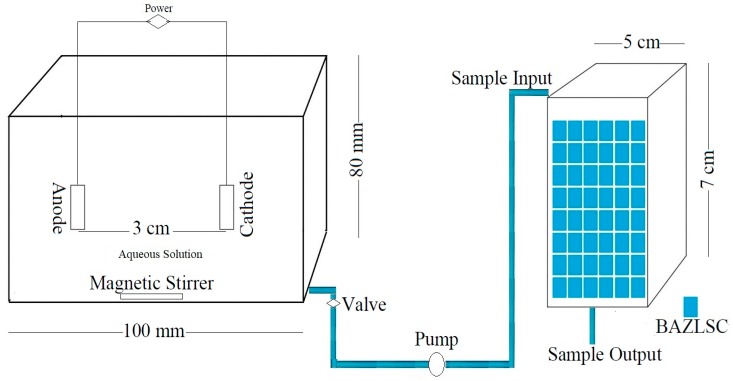
Schematic diagram of the EO reactor (**left**) and adsorption column (**right**) used in this study.

The treatment process in this study was divided into two stages. In the first stage, a synthetic aqueous solution was treated with EO. Statistical analysis and optimization were performed using response surface methodology (RSM). In the second stage, water was transferred to a fixed-bed adsorption column for further treatment. During this process, desorption isotherms were monitored. Figure 1 presents a schematic diagram of the reactor that was employed in this study.

### 2.1. Synthetic Aqueous Solution Production

To created synthetic polluted water, tap water was spiked with three compounds, ammonia (NH_3_-N), phenols, and Mo(IV). To create standard solutions of each contaminant, chemical-grade compounds were dissolved in water to the prescribed concentration. Aqueous ammonia was obtained by dissolving ammonium hydroxide in water [16]. The phenol had a purity of 99.9% and molecular mass of 94.11 g/mol based on laboratory analysis [17]. Finally, the standard solution of Mo(IV) was obtained by dissolving Na_2_MoO_4_·2H_2_O in water [18].

The pH of the influent solution was measured with a pH meter and was adjusted with 0.1 M NaOH or 0.1 M HCl.

### 2.2. EO Reactor Characteristics

A reactor with a working capacity of 500 mL, width of 100 mm, length of 100 mm, and height of 80 mm was used for EO. An electrical current was employed via a constant-voltage/current-controlled DC power source. Ti/RuO_2_–IrO_2_ electrodes were employed as the anode and cathode. A plate anode and plate cathode of the same dimensions (3.5 cm × 3 cm × 1 cm, L × W × T) were arranged parallel to each other. Based on preliminary experiments and Koppad et al. [19], who used a distance of 30 mm between the anode and cathode during wastewater treatment with EO, we set the distance between the anode and cathode as 30 mm. A magnetic stirrer was placed at the bottom of the reactor for mixing. The experiments were completed at room temperature. As an electrolyte, 1 g/L of Na_2_S_2_O_8_ added to the samples before each experiment [3]. The current and voltage were fixed at 50 mA and 7 V, and were set according to the ranges that were reported by Bashir et al. [3] and Mojiri et al. [12], respectively.

### 2.3. Statistical Analysis

We computed the elimination effectiveness using Equation (1).

(1) $$\mathrm{Removal}\;(\%)=\frac{(C_{i}-C_{f})\;100}{C_{i}}$$

where *C_i_* and *C_f_* denote the preliminary and final concentrations of the contaminants, respectively.

A three-level factorial design was created with Design Expert ver. 10.0.7 software for the experimental design and data analysis. The three independent factors in this research were reaction time (1, 2, and 3 h), initial contaminant concentration (10, 30, and 50 mg/L), and pH (3, 4.5, and 6). Ammonia, phenols, and Mo(VI) removal were selected as the response parameters. We performed preliminary experiments to narrow the ranges of the variables before carrying out the full factorial experiments, which were based on previous studies. For example, Kearney et al. [20] set the pH to 4 for ammonia elimination by EO. Meanwhile, Asghar et al. [21] reported that a 1-h contact time was optimal to treat an aqueous solution by EO. Finally, Xu et al. [22] reported that a 4-h contact time was optimal for the removal of cyanide from water by EO. In addition, we considered increased and decreased ranges to improve the accuracy of the results. The three independent factors and their corresponding levels are presented in Table 1. Equation (2), which is an empirical second-order polynomial model, considers the performance of the scheme.

(2) $$Y=\beta_{0}+\sum_{j=1}^{k}\beta_{j}X_{j}+\sum_{j=1}^{k}\beta_{jj}X_{j}^{2}+\sum_{i}\sum_{<j=2}^{k}\beta_{ij}X_{i}X_{j}+e_{i},$$

where *Y* signifies the response; *X_i_* and *X_j_* denote the variables; *β*_0_ is a fixed coefficient; *β_j_*, *β_jj_*, and *β_ij_* denote the interface coefficients of the linear, quadratic, and second-order terms, respectively; *k* denotes the quantity of considered factors; and, *e* denotes the error. We used analysis of variance (ANOVA) to analyze the results using Design Expert.

We selected the initial concentrations of pollutants based on preliminary experiments and a literature review. For example, Li et al. [23] used 25 mg/L as the initial ammonia concentration during the application of an electrochemical ion-exchange reactor for ammonia removal, while Peings et al. [24] used 30 mg/L as the initial phenol concentration for the removal from an aqueous solution by advance oxidation.

### 2.4. Fixed-Bed Adsorption Column

After treating the synthetic aqueous solution with an EO reactor, the samples were transferred to an adsorption column with a water pump for further treatment.

#### 2.4.1. Fixed-Bed Adsorption Column Preparation

Dynamic adsorption was performed in a 5–7-cm glass column filled with a 1-mm composite adsorbent, BAZLSC. Based on preliminary experiments, the contact time of water with the adsorption column was set to 10~15 min [25].

#### 2.4.2. Composite Adsorbent (BAZLSC) Preparation

**Figure 2:**
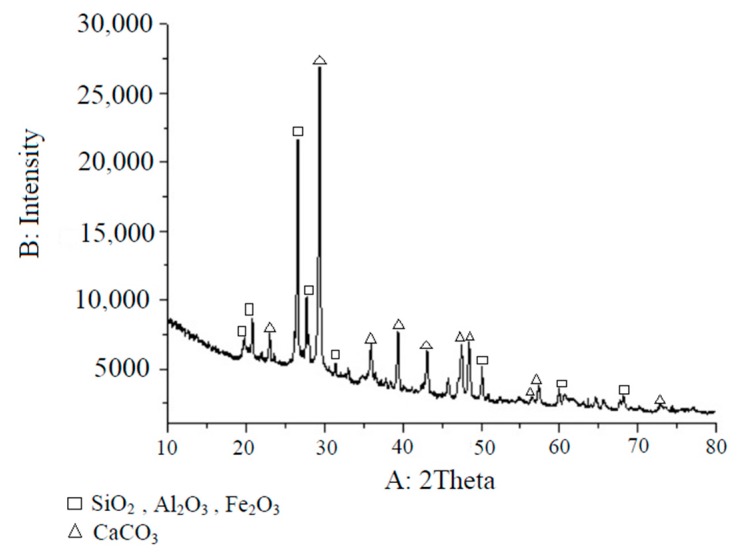
X-ray diffraction results of BAZLSC.

Bentonite, limestone, zeolite, cockleshell, activated carbon, and Portland cement were crushed, passed through a 300-µm mesh sieve, and then blended to obtain BAZLSC. The mixture was then carefully poured into a mold after adding water. The materials were removed from the mold after 24 h and immersed in water for approximately two days for the curing procedure. After letting the materials dry for three days, they were ground and passed through a sieve. Before using BAZLSC in the experiments, it was dried at 105 °C for 24 h. Table 2 and Figure 2 show the structures of the BAZLSC and x-ray diffraction analysis results, respectively. BAZLSC supported simultaneous adsorption and ion exchange [11].

#### 2.4.3. Adsorption Isotherm

Adsorption is the adhesion of atoms, ions, biomolecules, or molecules of gas, liquid, or solids onto a surface. The adsorption isotherm equation is (Equation (3)):

(3) $$q_{e}=\frac{(C_{0}-C_{e})V}{M},$$

where *q_e_* represents the sum of the solute adsorbed per unit weight of the adsorbent (mg/g), *C*_0_ denotes the preliminary adsorbate concentration, *C_e_* denotes the equilibrium adsorbate concentration (mg/L), *V* denotes the volume of the solution (L), and *M* represents the mass of the adsorbent (g).

The Langmuir isotherm represents the foundation of a monolayer adsorbate on the outward surface of an adsorbent. Hence, this isotherm represents the equilibrium spreading of ions between the solid and liquid phases [26]. Meanwhile, the Langmuir isotherm is suitable for monolayer adsorption onto a surface comprising a confined quantity of identical positions. The Langmuir equation can be expressed as Equation (4) [27]:

(4) $$\frac{x}{m}=\frac{abC_{e}}{\left(1+bC_{e}\right)},$$

where *x*/*m* denotes the mass of the adsorbate adsorbed per unit mass of adsorbent (mg/g), *a* and *b* denote the empirical fixed, and *C_e_* is the equilibrium concentration of the adsorbate in the solution after adsorption (mg/L).

The Freundlich isotherm is regularly applied in order to justify the adsorption characteristics of heterogeneous surfaces [26]. The Freundlich equation can be expressed as Equation (5):

(5) $$q_{m}=K_{f}C_{e}^{1/n},$$

where *K_f_* is a constant representative of the relative adsorption capability of the adsorbent (mg^1−(1/n)^ L^1/n^ g^−1^) and n denotes a constant that is related to the adsorption intensity [28].

Freundlich and Langmuir isotherms were used to simplify the characteristics of BAZLSC adsorption. The adsorption isotherms were monitored in batch experiments. First, 20 mg/L of each contaminant were added to 200-mL beakers containing various concentrations (0–2.5 g/L) of adsorbent. Then, the beakers were shaken at 200 rpm for 30 min [12].

### 2.5. Analytical Methods

We followed the Standard Methods for the Investigation of Water and Wastewater [29] to analyze wastewater. A YSI 556 MPS (YSI Inc., Yellow Springs, OH, USA) was employed to record the temperature (°C), pH, electrical conductivity (mS/cm), oxidation–reduction potential (mV), and salinity (g/L). Inductively coupled plasma optical emission spectrometry (Varian 715; Varian Inc., Palo Alto, CA, USA,) and a spectrophotometer (HACH/2500; HACH, Loveland, CO, USA) were used to measure the components of the water. Phenol was tested using a HACH DR/2500 based on method 8047 and method 2540B, the 4-aminontipyrine method. Ammonia was tested by using a HACH DR/2500 that is based on method 8190, the Nessler method.

## 3. Results and Discussion

**Figure 3:**
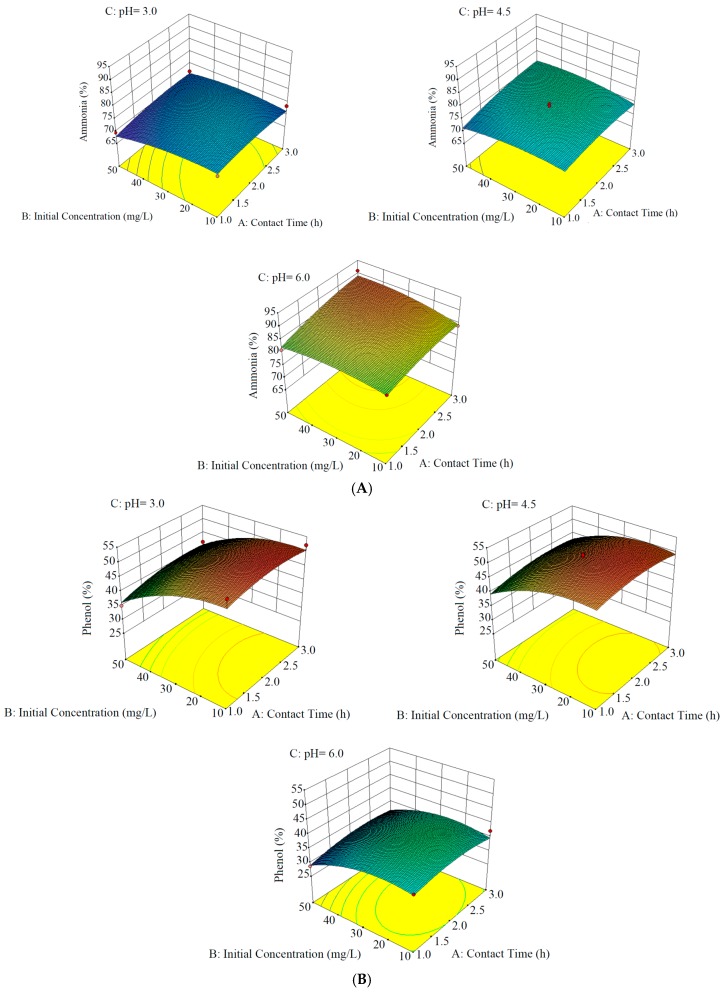

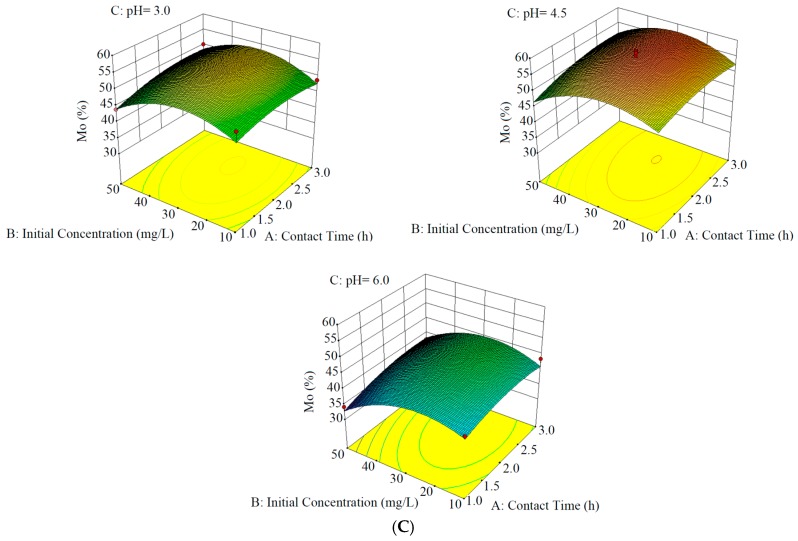
Three-dimensional surface plots of (**A**) ammonia, (**B**) phenol, and (**C**) Mo removal.

We investigated the elimination of ammonia, phenols, and Mo from contaminated wastewater via a combined EO and adsorption method. Table 1 and Table 3 show the independent variables of the three-level factorial design and the response values for the experimental conditions, respectively. Table 4 presents the statistical results of the response parameters. Figure 3 displays three-dimensional surface plots of contaminant elimination.

### 3.1. Ammonia, Phenol, and Mo Removal Using EO

The lowest elimination efficacy for ammonia was 69.5% (reaction time = 1 h, pH = 3, preliminary ammonia concentration = 50 mg/L) and the highest was 90.9% (contact time = 3 h, pH = 6, preliminary ammonia concentration = 50 mg/L) (Table 3 and Figure 3). Under the optimum conditions (contact time = 3 h, pH = 6, preliminary ammonia concentration = 44.8 mg/L), ammonia removal could reach approximately 91.0%. He et al. [30] investigated ammonia removal by EO at pH = 6.5 in the presence of Ru–Ir/TiO_2_ and Na_2_SO_4_. They expressed that up to 80% of ammonia could be eliminated and be mostly transferred to N_2_ in a powdered activated carbon (PAC) packed bed reactor under optimum conditions (pH = 6.5, I = 0.9 A, 2% Na_2_SO_4_, Cl^–^ = 1500 mg/L, and inlet velocity = 0.8 L/h). In addition, Ding et al. [31] removed 90% of ammonia using Ti/RuO_2_-Pt electrodes by the EO method. Similarly, Li et al. [23] reported an 89% ammonia removal at an initial concentration of 30 mg/L using a vermiculite-packed electrochemical reactor. Overall, the ammonia elimination efficiency in the current study was similar to those in previous studies.

The lowest phenol elimination efficacy was 28.7% (reaction time = 1 h, pH = 6, preliminary phenol concentration = 50 mg/L) and the highest was 52.1% (contact time = 3 h, pH = 3.5, initial phenol concentration = 10 mg/L) (Table 3 and Figure 3). Under the optimum conditions (contact time = 1.8 h, pH = 3, initial phenol concentration = 17.7 mg/L), the phenol elimination rate was approximately 52.6%. Saratale et al. [32] explored phenol elimination from wastewater by EO and reported an acidic pH and a voltage of 5 V for optimum phenol elimination. In addition, Wu et al. [33] investigated phenol removal by EO using N_2_SO_4_ as an electrolyte. Meanwhile, Tasic et al. [34] used two EO methods (direct and indirect oxidation) for the removal of organic contaminants. Direct oxidation of contaminants first leads to their adsorption onto the anode surface without the contribution of other substances in the solution, except for electrons, which are considered to be pure reagents. Direct electro-oxidation is ideally possible at low potential values before oxygen evolution, but the reactions are often slow and dependent on the electrocatalytic activity of the anode. Rapid electrochemical reaction is achieved while using noble metals and anodes that are based on metal oxides (e.g., IrO_2_, TiO_2_-Ru, and Ir-TiO_2_) for phenol elimination using the electrochemical technique. Two responses are probable for the direct anodic oxidation of organic pollutants [35].

(a) Electrochemical conversion, where the organic complexes are partly oxidized, according to the reaction (Equation (6)):
R → RO + e^−^ (6)
(b) Electrochemical combustion, where the organic complexes break down into CO_2_, water, and other inorganic complexes (Equation (7)):
R → CO_2_ + H_2_O + Salt + e^−^ (7)

The lowest elimination effectiveness for Mo was 34.1% (reaction time = 1 h, pH = 6, initial Mo concentration = 50 mg/L) and the highest was 59.4% (contact time = 2 h, pH = 4.5, initial Mo concentration = 30 mg/L) (Table 3 and Figure 3). Under the optimum conditions (contact time = 2.4 h, pH = 4.1, initial Mo concentration = 28.5 mg/L), the Mo removal rate was approximately 59.5%. Tran et al. [36] investigated metal elimination from an aqueous solution by the EO method at a voltage of 10 V. Their investigation showed a high performance in removing metals, but the selected voltage (10 V) and reaction time (20 h) were higher than the our findings.

In electrochemical treatments, the two methods for the elimination of contaminants in the presence of Na_2_S_2_O_8_ (as an electrolyte) are as follows:

(i) Direct oxidation, where metal cations (commonly heavy metals) are reduced at the cathode and organic contaminants are oxidized at the anode even without the connection of other chemical reagents [37].
(ii) Indirect electrolysis, where the concentration of Na_2_S_2_O_8_ hastens the mineralization of organic compounds. In general, reasonable concentrations of Na_2_SO_4_ accelerate the mineralization of organic matter via indirect oxidation, as shown in the following reactions [38]; however, it should be mentioned that some researchers have applied heat or ultraviolet light + heat in order to improve the persulfate oxidation ability of phenols [39].

The probable reactions occurring at the anode, cathode, and in the bulk material are shown below (Equations (8)–(15)) [40].

At the anode (oxidation):

SO_4_^2−^ + 2OH^−^ ↔ SO_4_^2−^ + 2e^−^ + H_2_O (8)

SO_4_^2−^ ↔ S_2_O_8_ + 2e^−^ (9)

4OH^−^ ↔ O_2_ + 2H_2_O + 4e^−^ (10)

At the cathode (reduction):

H_2_O ↔ H^+^ + OH^−^ (11)

2H_2_O + 2e^−^ ↔ H_2_ + 2OH^−^ (12)

HSO_4_^−^ + OH° ↔ SO_4_^−^° + H_2_O (13)

SO_4_^−^° + SO_4_^−^° ↔ S_2_O_8_^2−^ (14)

Organics + S_2_O_8_^2−^ → intermediates → H_2_O + CO_2_ + ↑A° (15)

Figure 3 shows the pollutant removal under different pH conditions, initial pollutant concentrations, and contact times, and has been extracted based on the information in Table 3 and Table 4. Figure 3A shows the effect of the operational parameters on ammonia elimination. Ammonia elimination increased with increasing initial concentration, pH, and contact time. With an influent pH of 6, initial concentration of 50 mg/L, and contact time of 90 min, the maximum ammonia removal was 90.95%. Figure 3B shows the effects of the variables on phenol elimination. Phenol elimination increased with an increasing initial concentration until 30 mg/L, pH until 3, and contact time until 2–3 h. Finally, Figure 3C displays the effects of the independent factors on Mo elimination. Mo elimination increased with increasing initial concentration until 30 mg/L, pH until 4.5, and contact time until 2 h.

### 3.2. Energy Consumption (EC; kWh/kg N)

EC (kWh kg^−1^ N) at optimum condition was calculated by Equation (16) [41]. EC states to the electrochemical treatment cost. The EC value was 8.0 (kWh kg^−1^) at the optimum operating conditions. Christiaens et al. [41] reported EC = 13.9 (kWh kg^−1^) during electrochemical ammonia recovery. EC in this contemporary research is lower than them, it displays the combined system could diminish energy consumption.

(16) $$\mathrm{EC}=\frac{UIt}{(N_{0}-N_{t})V},$$

where, *N*_0_ and *N_t_* present *N* at initial time and set time; *U*, *I* and *t* are voltage, current (A), and time (h), respectively; and, *V* is volume (L).

### 3.3. Ammonia, Phenol, and Mo Removal Using an Adsorption Column

The synthetic aqueous solution was initially treated in the EO reactor under the optimal conditions before being transferred to an adsorption column for the second stage of treatment. The performance of EO combined with adsorption enhanced the elimination efficiencies of ammonia, phenols, and Mo from 79.4% to 99.9%, 48.0% to 99.9%, and 55.9% to 99.9%, respectively. The composite adsorbent used in this study simultaneously performed adsorption and ion exchange, and it was produced from effective, low-cost materials, such as bentonite, zeolite, activated carbon, cockleshell, cement, and limestone. For example, Mazloomi and Jalali [42] reported that zeolite can be used to eliminate NH_4_^+^ from domestic and industrial wastewater. Meanwhile, Halim et al. [14] reported that activated carbon is effective in removing ammonia. Finally, Haseena et al. [43] investigated the potential use of bentonite in eliminating ammonia from aqueous solutions. Based on the literature [44], we speculated that bentonite and zeolite could be used to remove phenols and metals from water, which was verified by the results. As noted above, the composite adsorbent BAZLSC can perform ion exchange and adsorption, since it contains bentonite, zeolite, limestone, shell, cement, and activated carbon [45]. This has been confirmed in equilibrium and adsorption studies of composite adsorbents [46].

### 3.4. Adsorption Isotherms of Pollutant Removal by the Composite Adsorbent

**Figure 4:**
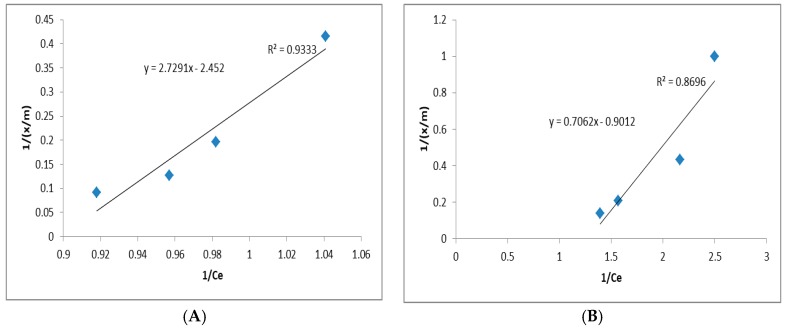

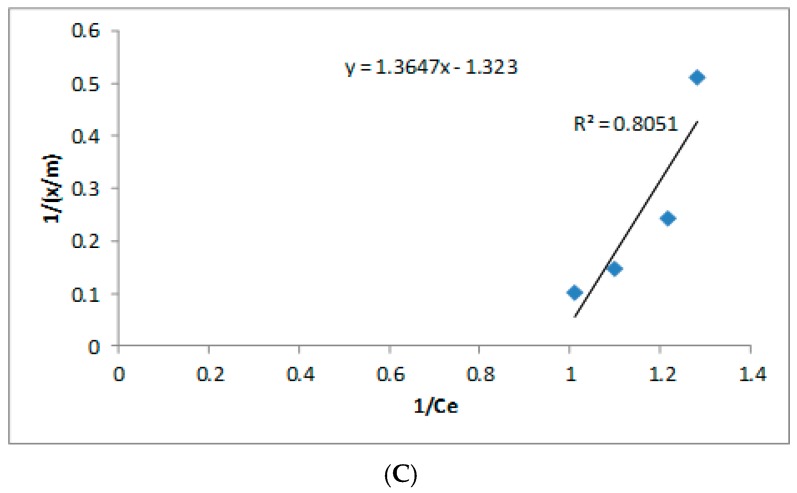
Langmuir isotherm regressions for (**A**) ammonia, (**B**) phenols, and (**C**) Mo.

Table 5 and Figure 4 show the Langmuir equation and isotherm regression for ammonia, phenols, and Mo, respectively.

The adsorption capacities (Q, Table 5) of ammonia nitrogen, phenols, and Mo were 6.198, 3.86, and 5.44 mg/g, respectively. Based on a study by Mojiri et al. [7], we determined that the composite adsorbent could reach Q = 0.70 mg/g for Fe adsorption. The energy of adsorption (b, Table 5) for ammonia nitrogen, phenol, and Mo removal were 0.240, 0.087, and 0.045 L/mg, respectively. By increasing the efficacy of elimination (i.e., increased *C_e_* values), the value of *x*/*m* decreased [4]. Finally, the regression coefficient (R^2^, Table 5 and Figure 4) was 0.9333, 0.8696, and 0.8051 for ammonia, phenols, and Mo, respectively. Similar values for R^2^ have been reported for ammonia by zeolite, activated carbon, and composite adsorbent [14]. Moreover, similar R^2^ values have been reported for phenol adsorption using granular activated carbon and Mo adsorption while using Pb–Fe-based adsorbent [47].

**Figure 5:**
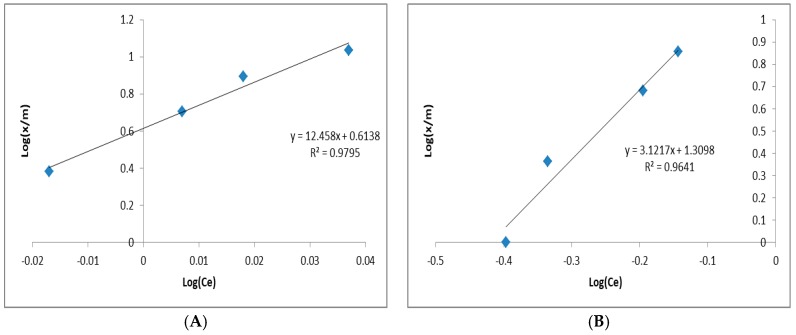

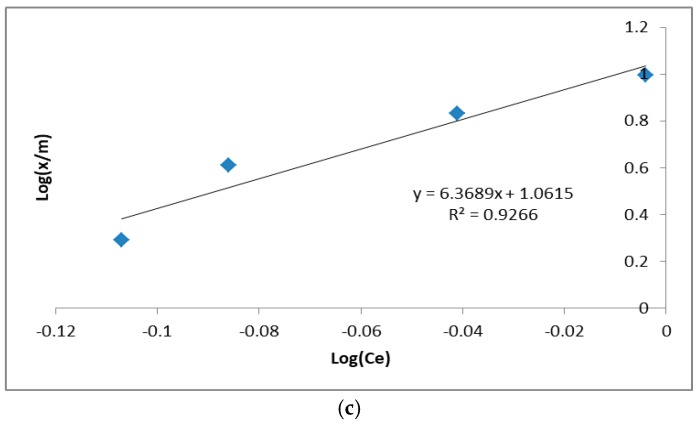
Freundlich isotherm regressions for (**A**) ammonia, (**B**) phenols, and (**C**) Mo.

The Freundlich capability factors (*K_f_*, Table 6) for ammonia, phenol, and Mo elimination were 0.014, 0.063, and 0.028 (mg/g (L/mg)^1/n^), respectively. The R^2^ values were 0.9795, 0.9641, and 0.9266 for ammonia, phenols, and Mo, respectively (Table 6 and Figure 5). These collective results indicated that the Freundlich isotherm is more suitable than the Langmuir isotherm for describing ammonia, phenol, and Mo removal by the adsorption column. Finally, greater capacities for adsorption are indicated by higher *K* values [7]. The collective 1/n values for ammonia, phenol, and Mo were 12.458, 3.1217, and 6.3689, respectively.

## 4. Conclusions

In this study, we successfully removed ammonia, phenols, and Mo from aqueous solution via combined EO and adsorption column. RSM was used to design the experiments. The key outcomes of this research indicate that the EO reactor could remove 79.4%, 48%, and 55.9% of ammonia, phenols, and Mo, respectively, under optimum conditions (contact time = 2.4 h, pH = 4.9, initial pollutant concentration = 27.4 mg/L), with a fixed current and voltage of 50 mA and 7 V, respectively. Next, the water was moved to an adsorption column filled with a composite adsorbent, BAZLSC. Adsorption improved the removal efficiency to 99.99% for ammonia, phenols, and Mo(VI). Finally, adsorption isotherm analysis revealed that the adsorption of ammonia, phenols, and Mo by BAZLSC followed the Freundlich isotherm equation better than the Langmuir isotherm equation. More investigation about using different adsorbents, different electrolytes, and electrolytes function would be considered in future studies.

## Figures and Tables

**Table 1:** Independent variables of the three-level factorial design.

Level	Reaction Time (h)	Initial Pollutant Concentration (mg/L)	pH
−1	1	10	3
0	2	30	4.5
+1	3	50	6

**Table 2:** Powdered BAZLSC characteristics.

Characteristic	Value
Surface area (m^2^/g)	288.6
External surface area (m^2^/g)	246.7
Micropore area (m^2^/g)	61.9
Micropore volume (cc/g)	0.08

**Table 3:** Response values under different experimental conditions.

Run	Contact Time (h)	Initial Concentration (mg/L)	pH	Ammonia Rem. * (%)	Phenols Rem. (%)	Mo Rem. (%)
13	0.8	30.0	4.5	74.17	45.17	49.64
6	1.0	10.0	3.0	72.11	51.95	49.87
8	1.0	10.0	6.0	82.62	35.12	40.13
4	1.0	50.0	3.0	69.53	35.11	44.00
11	1.0	50.0	6.0	81.11	28.71	34.18
17	2.0	6.0	4.5	74.31	44.11	46.11
2	2.0	30.0	4.5	77.00	50.86	58.95
3	2.0	30.0	6.3	90.11	33.00	38.00
5	2.0	30.0	2.7	71.17	43.84	47.11
9	2.0	54.0	4.5	72.00	42.92	47.11
10	2.0	30.0	4.5	76.92	49.97	58.74
12	2.0	30.0	4.5	77.11	50.81	59.49
15	2.0	30.0	4.5	77.12	50.57	58.00
16	2.0	30.0	4.5	77.00	50.89	58.76
18	2.0	30.0	4.5	76.93	51.18	59.00
20	2.0	30.0	4.5	76.93	51.11	59.40
22	2.0	30.0	4.5	77.71	50.35	59.10
7	3.0	10.0	6.0	84.13	37.19	43.45
21	3.0	10.0	3.0	73.46	52.17	48.69
14	3.0	50.0	3.0	71.64	41.50	50.13
1	3.0	50.0	6.0	90.95	29.97	37.64
19	3.2	30.0	4.5	73.18	46.18	53.90

* Abbreviation: Rem. means removal.

**Table 4:** Results of the analysis of variance of the response parameters.

Response	Final Equation in Terms of Actual Factor ^a^	R^2^	Adj. R^2^	Adec. P.	SD	CV	PRESS
Ammonia	94.27 + 1.032A − 12.849C + 1.655C^2^	0.9422	0.8988	18.19	1.80	2.40	334.32
Phenols	3.532 − 0.039B + 21.99C − 0.008B^2^ − 3.003C^2^	0.9297	0.8769	11.68	2.72	6.93	668.22
Mo(VI)	−22.835 + 30.369C − 0.013B^2^ − 3.634C^2^	0.9202	0.8604	11.98	3.06	6.65	712.52

Abbreviations: R^2^: Coefficient of determination; Adj. R^2^: Adjusted R^2^; Adec. P.: Adequate precision; SD: Standard deviation; CV: Coefficient of variation; PRESS: Predicted residual error sum of squares; ^a^ In the final equations, A is the electrochemical oxidation reaction time (h), B is the initial concentration of pollutants (mg/L), and C is pH; Significant at 0.05.

**Table 5:** Langmuir equation for ammonia, phenols, and Mo.

Parameter	Q (mg/g)	b (L/mg)	R^2^
Ammonia	1.027	0.240	0.9333
Phenols	0.554	0.087	0.8696
Mo	0.874	0.45	0.8051

**Table 6:** Freundlich equation for ammonia, phenols, and Mo.

Parameter	*K_f_* (mg/g (L/mg)^1/n^)	1/n	n	R^2^
Ammonia	0.014	12.458	0.080	0.9795
Phenols	0.063	3.121	0.320	0.9641
Mo	0.028	6.368	0.157	0.9266
